# Supplementary material for: Crossing the threshold of ultrafast laser writing in bulk silicon
Source: Nat Commun. 2017 Oct 3;8:773. doi: 10.1038/s41467-017-00907-8 (PMC5626724; doi:10.1038/s41467-017-00907-8)
Supplement: Supplementary file 1 — Supplementary Information [file 41467_2017_907_MOESM1_ESM.pdf]

Supplementary Note 1: Surface modification threshold

The majority of the experiments were performed with the femtosecond laser beams focused inside the bulk of silicon samples (at a depth of 1 mm). As conventional focusing configurations with ultrafast lasers do not allow to induce any modification in the bulk, we have first determined the fluence threshold for surface modification with the same laser beam focused at the surface of the same silicon samples. This serves as a guideline by comparison with the delivered fluences in the bulk interaction experiments.

The laser used in the experiments is detailed in the Methods section of the main manuscript. To determine the surface modification threshold, a silicon sample is repeatedly irradiated with single femtosecond laser pulses (focused with NA=0.3 objective) at gradually increasing pulse energies, all on fresh sites of the sample (different locations separated by 50 µm). The energy of the applied pulses is measured with a Joulemeter (GENTEC QE25-LP-H-MB-D0). To optimize the sample surface positioning (at the best beam focus; the minimum spot size and maximum intensity), the experiment is repeated applying a z-scan procedure where the position of the focus is changed along the optical axis with 10-µm increments (by moving the focusing objective on a motorized stage). The results of the experiment leading to modifications with the lowest energy threshold (best focus ± 5 µm) are then analyzed in details. To do so, the sample is observed with a bright field reflection microscope (ZEISS Axiotec) and for each irradiated spot we determine if a modification can be detected by inspection with 20× magnification.

To report a fluence threshold, the beam focus is fully characterized in air using rigorously the same 3D fluence imaging procedure as that applied in silicon and discussed in the manuscript. As shown in Supplementary Figure 1, we find that the best focus exhibits a nearly Gaussian energy distribution (vertical profile, right graph) with a waist $w_0 \cong 2$ µm ($\cong 2.4$ µm full width at half maximum). Accordingly, we associate the measured pulse with an average fluence simply given by $F = E/\pi w_0^2$ where E is the pulse energy. There are two main sources of experimental uncertainty for the delivered fluence in this experiment. First, is the laser fluctuation ΔF_{las} of about 4% (rms), as determined with the Joulemeter. Second, is the precision when positioning the focus at the surface. With the employed z-scan procedure, the precision for focus positioning is ± 5µm. According to the 3D fluence mapping, shown in Supplementary Figure 1, this causes another uncertainty $\Delta F_{pos.}$ of ± 5% on the estimated delivered fluence. The combined errors of about ± 9% are shown with horizontal bars in Supplementary Figure 2 reporting the observation of modifications as a function of the laser fluence.

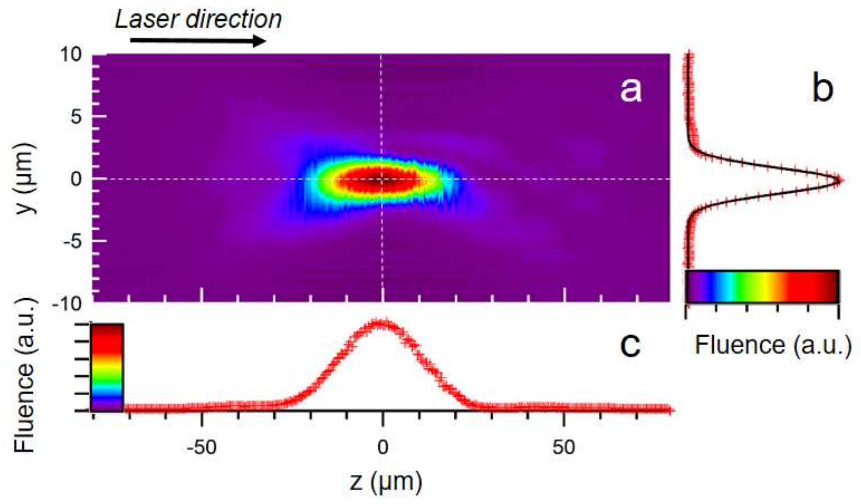

Supplementary Figure 1 | Cross-sections of the measured fluence distribution at focus in air. (a) Two-dimensional cross-section along the optical axis. (b) and (c) are respectively the vertical and horizontal fluence profiles along the white dotted lines shown in (a). The measurements (red symbols) are compared with a theoretical Gaussian profile of 2.4 µm full width at half maximum (solid black). This measurement is used for the determination of the applied laser fluences in the bulk and for the surface damage study (calibrations).

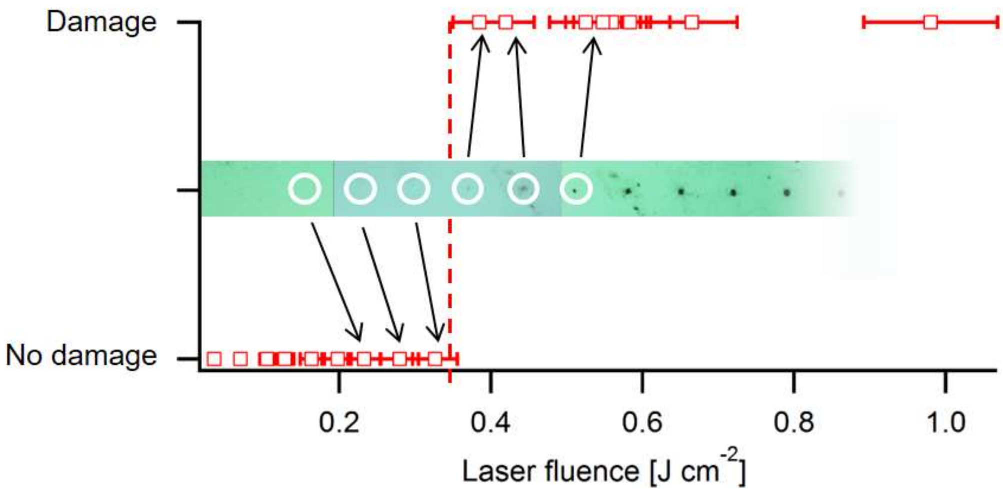

Supplementary Figure 2 | Determination of the fluence threshold for silicon surface modification with 60-fs laser pulses at 1300 nm. A sample is repeatedly irradiated with single pulses (focused with NA=0.3 objective) at gradually increasing pulse energies, all on fresh sites of the sample. As illustrated by the white circles on several microscopy images gathered in the green region, each site is inspected for a damage / no damage determination as a function of fluence. The error bars show the systematic 9% error on the determination of the applied fluences and accounting for the positioning accuracy of the focus and the laser energy fluctuations. The threshold is defined as the fluence above which a modification is systematically detected, that is 0.35 J cm^{-2}.

With this experiment, we estimate that the fluence threshold for silicon surface modification is $F_{th} \cong 0.35 \pm 0.03$ J cm^{-2} with our 60-fs laser pulses at 1300-nm. This is consistent with values reported in the literature[1,2]. The precision of this estimate could be easily improved with a statistical study on the occurrence of modifications for near-threshold conditions. However, we simply intend here to compare the delivered fluence in the bulk with the typical fluence for surface modification.

While we applied on the surface the same laser beams as for the bulk studies, we acknowledge that a direct one to one comparison is not straightforward, for two reasons. First, bulk modification thresholds are usually higher than surface thresholds[3]. Second, while the spatial and energy characteristics of the beams can be directly compared, it remains a difference in the pulse duration because of pulse broadening by group velocity dispersion (GVD) in the the bulk of silicon. For unchirped, transform-limited Gaussian pulses propagating in a material of thickness L, the broadened width (FWHM) is directly given by $\tau = \tau_0\sqrt{1 + (4\ln(2)\,\mathrm{GVD}\,L/\tau_0^2)^2}$ where τ_0 is the initial pulse duration[4]. With a GVD of $\cong$ 1645 fs^2 mm^{-1} for silicon at 1300 nm, we find that the 60-fs duration pulses used for surface interaction are transformed in 90-fs pulses at the focus when attempting modification at a depth of $L = 1$ mm below the surface of silicon. Obviously, we predict that a higher surface damage threshold would have been measured if 90-fs pulses were applied[5] and we likely underestimate the threshold that should serve for a more direct comparison with the bulk experiments.

For these reasons, we conclude that the measured fluence threshold can only be seen as a minimum target for the fluence to be delivered in the bulk of silicon in order to envision material modifications. We show in the main manuscript that focusing with hyper-NA values above 2.9 is required to deliver laser fluences at the focus exceeding the measured threshold of 0.35 J cm^{-2}.

Supplementary Note 2: Focusing and imaging in a Si-sphere

To overcome the NA limitation of conventional configurations, we have replaced the silicon slab samples by high-resistivity silicon spheres so that the wavefront curvature of the focused beam matches the air-silicon interface. By suppressing in this way the refraction as illustrated in Supplementary Figure 3, we apply a solid-immersion focusing strategy and we have access to extremely high NA values for both the interaction and imaging diagnostics.

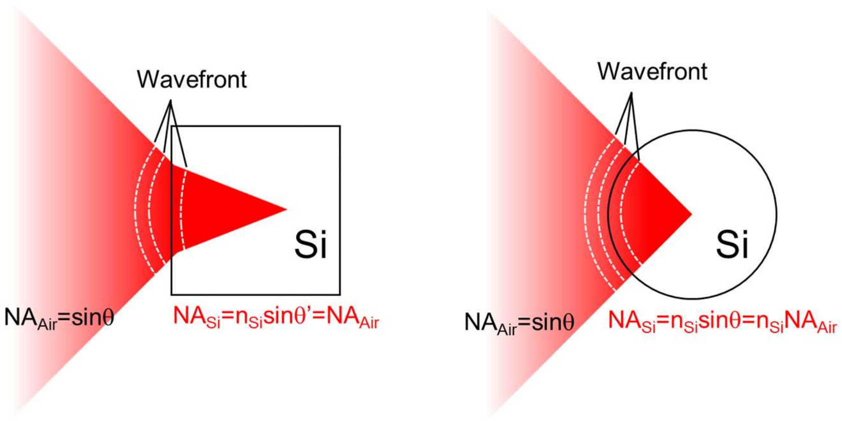

Supplementary Figure 3 | Simplified schematic of the applied strategy. When the radiation is focused at the centre of silicon spheres, the wavefront curvature shown by the white dotted lines matches the air-silicon interface suppressing its transformation due to refraction. In comparison to an experiment with a flat sample, this leads to: (1) a numerical aperture NA increased by a factor n_{Si}, the refractive index of silicon (2) an aberration-free spot (spherical, chromatic and coma aberrations) and (3) a magnification factor of n_{Si} when imaging the focus region.

Accessible Ultra-High Numerical-Apertures

By definition, the numerical aperture of an objective is defined by $\mathrm{NA} = n\ sin\theta$ where θ represents the maximum half-angle of the cone of light that exits the lens where $n = 1$ for air-standing objectives. In Supplementary Table 1, we list the NIR microscope objectives (Olympus LMPLN-IR/LPCPLN-IR lenses) used for the experiments. The lenses are specifically designed for NIR wavelengths and all exhibit a transmission >75% at 1300 nm. When focusing inside planar samples, the apparent NA is conserved because $\mathrm{NA} = n\ sin\theta$ is constant across an interface according to Snell's law. Then, it is striking to note in Supplementary Table 1 the very modest θ value of only 14.4° when focusing inside Si ($n_{\mathrm{Si}} = 3.5$) with the maximum NA=0.85 (instead of 60° in air). When focusing at the centre of spherical samples, all angular components of the beam are normal to the air-Si interface. Then, the spherical geometry suppresses refraction and θ is conserved across the air-Si interface while the refractive index is changed from 1 to 3.5. This leads to the increased effective NAs that are reported in the Supplementary Table 1 for all objectives. We note that an effective NA=2.97 is reached for our objective with the highest numerical aperture.

A major interest of this experimental strategy is to take benefit of high angular components so that intensities remain modest until the central core region of the focus is reached. Then, it is preferentially here that significant nonlinear absorption can occur. In addition, it is the far-field diffraction barrier that limits the minimum spot size to about half the processing wavelength in conventional configurations, imposing a serious restriction on the confinement of the interactions. By applying the hyper-NA strategy in spheres, one can expect a spot size $w_0 \approx \lambda/2\mathrm{NA} \cong \lambda/6$ of about 220 nm and a corresponding confocal parameter $b = 2Z_R = 2n_{\mathrm{Si}}\pi w_0^2/\lambda$ of about 820 nm. Then, we hold a situation with an improved geometry for the laser energy flux but also an intrinsic enhancement of the confinement of the interactions.

Model	LMPLN5XIR	LMPLN10XIR	LCPLN20XIR	LCPLN50XIR	LCPLN100XIR
Correction Si (mm)	-	-	0-1.2	0-1.2	0-1
Focus in air					
θ	5.7	17.5	26.7	40.5	58.2
NA	0.1	0.3	0.45	0.65	0.85
Focus in silicon samples with flat surfaces					
θ	1.6	5	7.6	11.5	16.6
NA	0.1	0.3	0.45	0.65	0.85
Focus at the center of silicon spherical samples					
θ	5.7	17.5	26.7	40.5	58.2
NA	0.35	1.05	1.57	2.27	2.97

Supplementary Table 1 | Focusing characteristics of the femtosecond laser beams in air, flat and spherical silicon samples. For each microscope objective identifiable by the model number (Olympus), we provide the maximum half-angle of the focused beam θ and effective numerical aperture NA when focusing the femtosecond laser pulses in air, inside flat silicon samples and at the centre of silicon spheres. We note that the objectives with NA > 0.45 (in air) are equipped with correction collars which allows to cancel spherical aberrations in all cases. The correction is systematically set at 0 thickness for air and Si spheres and at 1-mm thickness of Si for flat samples.

Suppression of aberrations

The absence of refraction at the air-material interface provides additional benefits, like the suppression of spherical and chromatic aberrations. The spherical symmetry obviously eliminates coma and the overall optical system including the material becomes aplanatic. This is an important aspect to ensure the highest level of confinement for the interactions but also for the imaging performance of the lateral microscopy diagnostics. In practice, the objectives with the highest NA values are equipped with correction collars that are adjustable according to the thickness of silicon or glass substrates (see table 1). For the experiments in Si samples with flat surfaces, the pulses are focused at a depth of 1 mm below the surface and the correction is systematically adjusted to compensate aberration. For the interactions at the centre of spherical samples, the correction is set to zero because of the intrinsic absence of aberration with the spherical symmetry. By this means, we are able to readily address the aberration issues and we deal with non-aberrated conditions in all the experiments.

Lateral magnification

When imaging the microplasmas and modifications at the centre of the sphere samples, the observations are magnified by a factor that can be easily calculated under the small angle approximation. Referring to Supplementary Figure 4, the object is denoted by the “o” subscript and the image is denoted by the “i” subscript. The object is placed at the centre of a sphere of radius R of refractive index n_s and surrounded by air (n = 1). Under the small angle approximation, we can assume $\theta_o \simeq h_o R$ and $\theta_i \simeq h_i R$ where h_o is the height of the object and h_i is the height of the image. Using Snell’s law, $n_s \sin\theta_o = \sin\theta_i$, which in the small angle approximation reads $n_s \theta_o = \theta_i$, we can access the lateral magnification of the object seen from air defined by $m \equiv h_i/h_o$ and given by $m = \theta_i / \theta_o = n_s$.

The small angle approximation holds for objects much smaller than the sphere (h_o<<R) and observations with modest NA objectives. In the experiments, R=1 mm and h_o is of micrometre dimension. Thus, the

first condition is fulfilled. The second is more questionable for high-resolution microscopy observations. To confirm the magnification experimentally, we imaged a calibration target with and without a hemispherical silicon sample of size similar (1-mm radius) to that of the spheres for the main experiments (same provider). The comparison is shown in Supplementary Figure 5 for observation with our NA=0.3/X10 objective. Under these conditions, we confirm the additional magnification factor of $n_s \cong 3.5$ (the refractive index of silicon) that must be taken into account for imaging at the centre of the spheres.

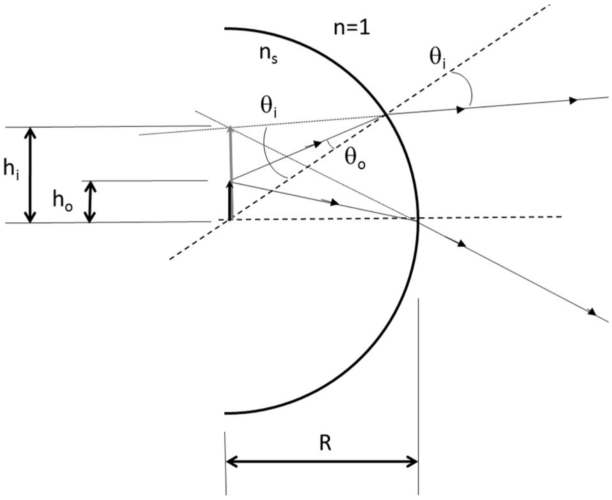

Supplementary Figure 4 | Magnification of observations at the centre of a sphere of radius R and refractive index n_s. Taking a ray of incidence angle θ_o emerging form an object of height h_o at the centre of the sphere, we reveal how the refraction at the sphere interface transform the angle in θ_i leading to a magnified image of height h_i at the same location as the object. Under the small angle approximation, we obtain a magnification directly given by n_s the refractive index of the sphere.

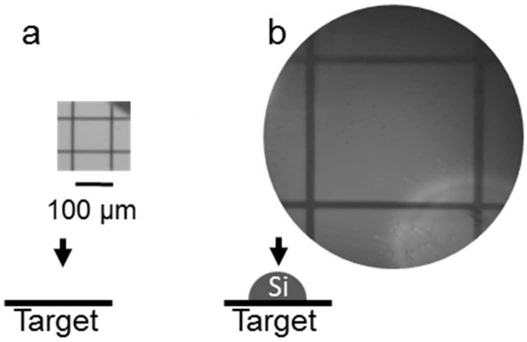

Supplementary Figure 5 | Effect of the spherical interface on the infrared microscopy images. We show a direct comparison of images obtained by reflection microscopy of a calibration target (metal lines forming 100-µm edge squares) without (a) and with (b) a hemispherical silicon sample positioned on top of the target (imaging through the sphere). With the sphere, a lateral magnification factor of $n_{Si} \cong 3.5$ is directly observed.

Supplementary Note 3: Quantitative phase infrared imaging of the modifications

Experimental setup and methods

To characterize the refractive index changes associated with laser-induced modifications inside c-Si spheres, we have specifically transposed to the infrared domain of the spectrum a longitudinal-differential interferometry technique[6] for phase imaging. Our setup is presented in Supplementary Figure 6. It employs the same ultrafast laser for writing the modification and for coherent illumination in the phase imaging setup.

In reality, there is no need for ultrafast pulses for phase imaging of permanent modifications by an interferometric method. However, the short pulse duration of <100 fs offers the advantage to be associated to an effective coherence length that is below 30 µm that eliminates parasitic interferences of residual reflections from the optical components in the beam paths (tested comparison with a CW laser). Another advantage is the possibility of time-resolved observation of the nanosecond-lived microplasmas at low pump energy by simply implementing a delay line (DL in Supplementary Figure 6). While the quantitative analysis of the microplasmas[7] generated with hyper-focused pulses in the spheres could not be achieved due to resolution and sensitivity limits of our measurements, it remained useful for alignment purposes. In practice, to prepare the experiment, a plasma is first generated in air and observed with the microscopy setup. Then, the sphere is centred with respect to the position of the observed plasma and a plasma is observed at low energy (below breakdown threshold) inside the sphere. Then, we check that the position of the plasma in the Si sphere is the same as that in air, ensuring in this way that the sphere is precisely centred. This is a crucial step as both the focusing of the pump and the imaging inside the sphere is prone to aberration with misalignment of the sphere.

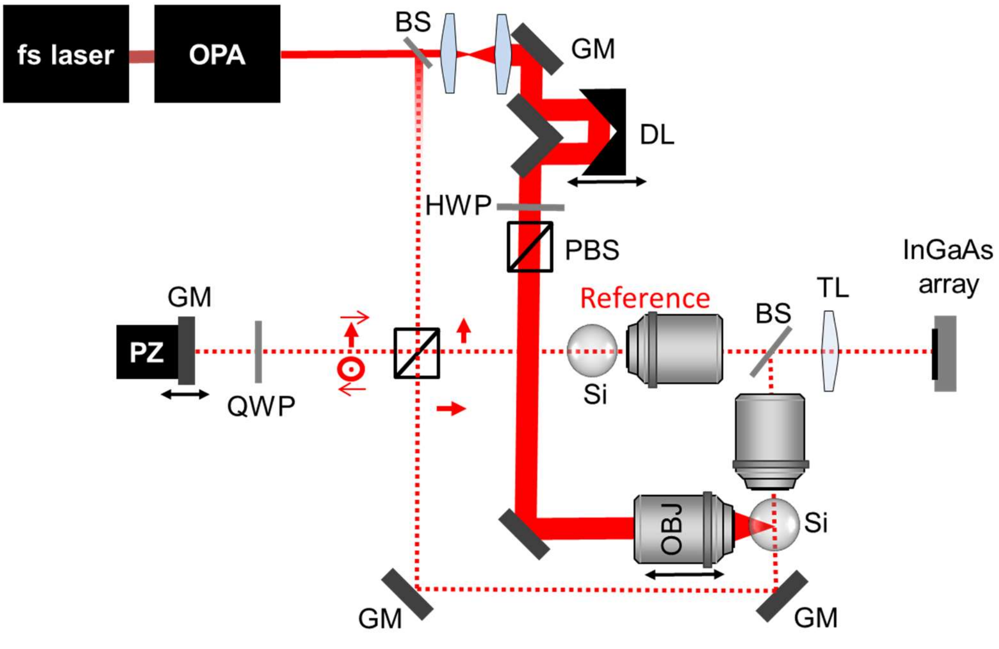

Supplementary Figure 6 | Schematic view of the experimental arrangement for quantitative-phase microscopy of modifications inside Si spheres. The femtosecond laser beam from the OPA (λ=1300 nm) is split in probe and pump beams with a beam splitter (BS). The energy of the pump pulses creating the modifications is controlled by a combination of a half-wave plate (HWP) and a polarizer. The probe beam (dotted line) is directed towards a phase shifting interferometry setup composed of a piezo-stage (PZ), a quarter-wave plate (QWP), polarizing beam splitters (PBS), objective lenses (OBJ), and a tube lens (TL). Bold red arrows indicate the polarization direction. The pump beam is blocked for investigations of permanent modifications but a delay line (DL), composed of gold mirrors (GM), can be adjusted for transient plasma observations.

The optical arrangement for imaging the modification (probe, see dotted lines in Supplementary Figure 6) takes the form of a Mach-Zehnder type interferometer. The probe beam is separated by a polarizing beam splitter (PBS). One of the beams (the reference beam in the upper arm of the interferometer) propagates through a quarter-wave plate (QWP) and is reflected by a gold mirror (GM) mounted on the piezoelectric stage (PZ) enabling nanometre precision motion along the optical axis. With two passes through the QWP, its polarization direction is rotated by 90 degrees and it is transmitted through the PBS. Accordingly, the beams in both arms of the interferometer exhibit the same polarization and interfere on the InGaAs array infrared-sensitive detector (Raptor, OWL SWIR 640, pixel size of 15 μm).

The object beam (lower arm of the interferometer) illuminates the sample (silicon sphere) which is imaged by a microscopy arrangement, composed of a microscope objective lens (OBJ; Olympus LCPLN20XIR) and its associated tube lens (TL), achieving about 70× magnification, taking into account the magnification factor of both the microscope (20×) objective lens and the spherical Si-air interface (3.5×). To avoid a large difference in the wavefronts between the object and reference beams on the detector, an identical sphere and microscope objective system is placed in the reference beam path. The tube lens (TL) is placed after the beam splitter (BS) which is used to recombine the beams so that it is shared by both beams.

With this arrangement, we record a microscopy image on the detector that is the result of the two-beam interference and its intensity distribution is given by the general form:

$$I(x,y) = A_O^2(x,y) + A_r^2(x,y) + 2A_O(x,y)A_r(x,y)\,cos[\Delta\phi(x,y)] \quad (1)$$

where $I(x,y)$ is the intensity distribution of the interference pattern in the plane of the array sensor (perpendicular to the optical axis). $A_\mathrm{o}(\mathrm{x,y})$ and $A_\mathrm{r}(\mathrm{x,y})$ correspond to the amplitude distribution of the object beam and reference beam in this plane, respectively and $\Delta\phi(\mathrm{x,y})$ corresponds to the phase difference between the object and the reference beams. Then, by moving the piezoelectric stage on the reference arm we can precisely control the relative phase between the two beams. The signal on each pixel is modulated and the relative phase difference between two regions of the image can be directly evaluated by plotting the intensity as a function of the optical path difference between the two beams (see traces A and B in Figure 3c of the main manuscript).

For a rapid retrieval of a complete phase difference image, we apply a simplified four-step procedure[8,9]. In this procedure, four interference images are acquired by making precise increments of $\lambda/8 = 162.5$ nm on the piezoelectric stage, so that a $\pi/2$ relative phase delay is added between acquisitions. An example of four interference images acquired on a modification at the centre of a Si sphere is shown in Supplementary Figure 7. Here, three modifications were previously written with 1000 laser pulses of 20-nJ energy with an effective NA of 2.97. According to the four-step procedure, the interference images can be formulated by the corresponding expressions:

$$I_k(x,y) = A_O^2(x,y) + A_r^2(x,y) + 2A_O(x,y)A_r(x,y)\,cos[\Delta\phi(x,y) + \frac{(k-1)\pi}{2}] \quad (2)$$

with $k = 1, 2, 3, 4$ and the quantitative phase image is directly given by[8]:

$$\Delta\phi = \arctan((I_4 - I_2)/(I_1 - I_3)) \quad (3)$$

The result of this phase retrieval from the four acquired interference images is shown in Supplementary Figure 7. A striking feature when comparing the images is the ability of phase microscopy to reveal details of the poorly contrasted modifications in the amplitude and interference images. This is because the image difference in Eq. (3) intrinsically eliminates all defects induced by imperfections seen by both beams.

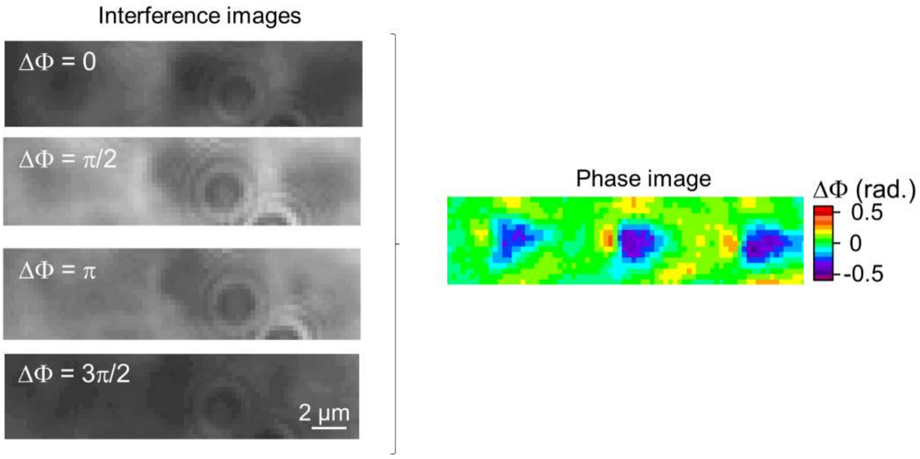

Supplementary Figure 7: Phase image reconstruction. Four phase-shifted interference patterns recorded by the phase microscopy and the corresponding phase data retrieved from the images. The modifications correspond to three laser-written regions near the centre of a Si sphere with the same writing conditions (1000 pulses, 20 nJ, NA=2.97).

Also, if phase imperfections (flatness, dust, etc..) are seen by only one beam, they can be readily suppressed in the phase image by subtracting a reference phase image before writing the modifications. For instance, one can note rings (likely due to dust on optics) on the interference images in Supplementary Figure 7 that are completely suppressed in the phase image.

Assuming an axial symmetry along the optical axis for the modifications, the phase image allows evaluating the refractive index modification according to

$$\Delta n = \Delta\phi\, \lambda \,/(2\pi\, d) \tag{4}$$

where $\Delta\phi$ and d are the apparent phase and diameter of the modification respectively. According to the apparent $\Delta\phi$ = -0.4 radians and $d \cong$ 1.2 µm, we observe a negative refractive index change near 0.7 for these experimental conditions. We established that smaller features produced with less applied pulses could be hardly characterized due the sensitivity limitation of our measurement. However, we have checked the repeatability of this typical index variation over more than 5 different modifications produced with the same laser conditions (1000 pulses, 20 nJ, NA=2.97).

Tests on a calibrated sample

According to the aforementioned four-step phase shifting procedure and associated equations, there is no need for a calibration of the instrument for quantitative phase measurements. However, we checked the validity and precision of the applied methodology by measuring a calibrated object. A thin film of silicon (25 nm thickness measured by atomic force microscopy) was deposited on a glass slide (thermal evaporation) with a mask so that the sample exhibits a Si stripe. By imaging this stripe with our infrared phase microscope, we confirmed the sign and the precision of our phase measurement. Also we checked the reproducibility by repeating an identical measurement 100 times (computer controlled automated procedure). We obtained a standard deviation on the measured phase-shift of about 7%, provided that each of the four interference images is averaged over 16 probe pulses, to reduce the contribution of laser fluctuations.

Supplementary Note 4: Nonlinear propagation study

Our experimental results are compared to nonlinear propagation simulations in order to identify the physical mechanisms responsible for the strict fluence clamping at a level that we raised above silicon breakdown threshold by using hyper-focused configurations.

Modelling equations

For the simulations we use the Unidirectional Pulse Propagation Equation (UPPE)[10] and apply the transformation optics approach fully described in ref.[11] to deal with the strongly nonparaxial nature of the problem. The UPPE takes the form:

$$\frac{\partial \hat{E}}{\partial z} = ik_z\hat{E} + i\frac{\mu_0\mu\omega^2}{2k_z}\hat{N} \tag{5}$$

where $\hat{E} = \hat{E}(\omega, k_x, k_y, z)$ is the spatio-temporal spectrum of the laser pulse, $k_z = [k^2(\omega) - k_x^2 - k_y^2]^{1/2}$ is the propagation constant, k_x, k_y and ω are the spatial and temporal angular frequencies, μ_0 and μ are the vacuum and medium permeabilities, respectively.

The last term in the right-hand side accounts for the nonlinear response including the third order nonlinear polarization P_{nl}, the current of free electrons J_f and the current that is responsible for multiphoton absorption J_a according to:

$$\hat{N} = \hat{P}_{nl} + \frac{i}{\omega}(\hat{J}_f + \hat{J}_a) \tag{6}$$

with

$$\hat{P}_{nl} = \frac{3}{4}\epsilon_0\chi^3\widehat{|E|^2E} \tag{7}$$

$$\hat{J}_f = \frac{{q_e}^2}{m_e}\frac{\nu_c + i\omega}{\nu_c^2 + \omega^2}\rho\hat{E} \tag{8}$$

$$\hat{J}_a = K\hbar\omega_0\frac{\delta\rho}{\delta t}\hat{E} \tag{9}$$

where $\hat{}$ denotes the spatio-temporal spectrum, ε_0 is the vacuum permittivity, $\chi^3 = 4n_0^2\epsilon_0 c_0 n_2/3$ is the cubic susceptibility with n_2 being the nonlinear index, n_0 is the medium refractive index at the pulse central frequency ω_0, c_0 is the speed of light in vacuum, q_e and m_e are the charge and mass of the electron, ν_c is the collision frequency, ρ is the density of free electrons (all in SI units) and K is the multiphoton order of ionization. In the expression for $\hat{P}_{nl}$ we neglect the effect of third harmonic generation. The real part of $\hat{J}_f$ describes inverse Bremsstrahlung absorption, and the imaginary part is responsible for plasma defocusing.

Together with the UPPE we solve the kinetic equation for plasma concentration:

$$\frac{\partial \rho}{\partial t} = \sigma_k I^k(\rho_{at} - \rho) + \sigma(\omega_0)\frac{I}{U_i}\rho \tag{10}$$

where $I = n_0\varepsilon_0 c_0|E|^2/2$ is the pulse intensity, ρ_{at} is the initial valence electron density, σ_K is the cross section of multiphoton ionization (corresponding to the multiphoton absorption cross section $\beta_K = K\hbar\omega_0\sigma_K\rho_{nt}$), U_i is the band gap and $\sigma(\omega_0)$ is the inverse Bremsstrahlung absorption cross section at the pulse frequency ω_0 given by

$$\sigma\,(\omega_0) = \frac{2\,{q_e}^2}{m_e n_0 \epsilon_0 c_0}\frac{\nu_c}{(\nu_c^2 + \omega_0^2)} \tag{11}$$

Also, because the avalanche ionization is a consequence of inverse Bremsstrahlung absorption accounted in $\hat{J}_f$, for the calculation of $\partial\rho/\partial t$ in $\hat{J}_a$ we use only the first term on the right-hand side of the kinetic equation for plasma concentration (Eq. (10)).

Accounted physical processes and parameters

According to the above described formalism, the model includes the following list of physical processes:

- Diffraction
- Dispersion
- Kerr cubic nonlinearity (without third harmonic generation)
- Defocusing in plasma
- Inverse Bremsstrahlung absorption
- Multiphoton absorption and ionization
- Avalanche ionization

The material parameters that we used in the simulations are presented in Supplementary Table 2.

	Parameter	Value	Units	Source
Refractive index	$n(\lambda)$			12
	n_0	3.51		12
Nonlinear refractive index	n_2	1.5×10^{-18}	$m^2\ W^{-1}$	13
Electron collision rate	ν_c	0.3×10^{15}	s^{-1}	14
Band gap	U_i	1.12	eV	
Multiphoton order	K	2		
Multiphoton absorption coefficient	β_K	0.8×10^{-11}	$m\ W^{-1}$	15
Atomic density	ρ_{at}	5×10^{28}	m^{-3}	

SupplementaryTable 2 | Material parameters for silicon at 1300-nm wavelength used in the simulations. The last column mentions the references from where the values are extracted.

The initial condition to solve the UPPE is a Gaussian beam in time and a truncated uniform spherical wave (flat top beam) in space:

$$E(t, x, y, z = 0) = \Theta(a - r)\exp\left[\frac{-(t + dt)^2}{2t_0^2}\right]\exp[-ik_0\frac{r^2}{2f} - i\omega_0 t] \tag{12}$$

where $k_0 = n_0\omega_0/c_0$, t_0 is the pulse duration taken at 60 fs. For the Gaussian temporal envelope, we introduce a spatially dependent temporal delay dt given by

$$dt = \frac{n_0}{c_0}\frac{r^2}{2f} \tag{13}$$

This term compensates the phase delay that appears after we apply the parabolic spatial phase and is required to describe a situation in which all angular components are synchronized at the focus[16]. For the spatial distribution, Θ is the step function, f is the focal distance and a the radius of the incoming beam which are fixed according to the numerical aperture given by $NA = n\, sin\theta = n\, a/f$ (see details in reference[11]). Figures 1 and 2 of the main manuscript show the results of nonlinear propagation simulations in Si for 60-fs laser pulses focused with NA from 0.3 to 1.5. In section 4.4, we provide more details on the simulation results at the maximum NA of 1.5.

Factors affecting the fluence delivered in Si

In our numerical studies we first focused on elucidating the origin of the fluence clamping, which is observed experimentally in Si. For simplicity, we consider only NA=0.45 in these investigations. Figure 1b of the main manuscript shows cross-sections of the 3D fluence distribution near the focus for different incoming pulse energies. For each case, we now compare two laser pulse energies: 10 nJ and 500 nJ, which are respectively near and far above the energy required to observe the saturation of the delivered fluence (see Fig. 2a of the main manuscript). Results are presented and discussed in terms of peak laser fluence, peak plasma density and pulse energy as a function of distance along the optical axis (z=0 mm corresponding to the air-Si entrance interface).

Kerr nonlinearity

Supplementary Figure 8 shows the results of simulations in which the Kerr nonlinearity is arbitrarily switched off ($n_2 = 0$). At low energy there are only very modest differences in peak fluence and plasma density with the original simulation. Also, we observe almost no displacement of the maxima on the optical axis. At high energy we see slightly smaller peak fluence and almost half the plasma density. At 500 nJ the critical power (≅ 24 kW) is largely exceeded and self-focusing is obviously expected. If the Kerr nonlinearity is switched off, fewer electrons are required to balance the focusing leading to a decrease of the apparent peak fluence. Accordingly, we conclude that the strong Kerr nonlinearity of Si (100 times higher than that of dielectrics, like silica) is not a limiting factor to the delivered fluence in the experiments.

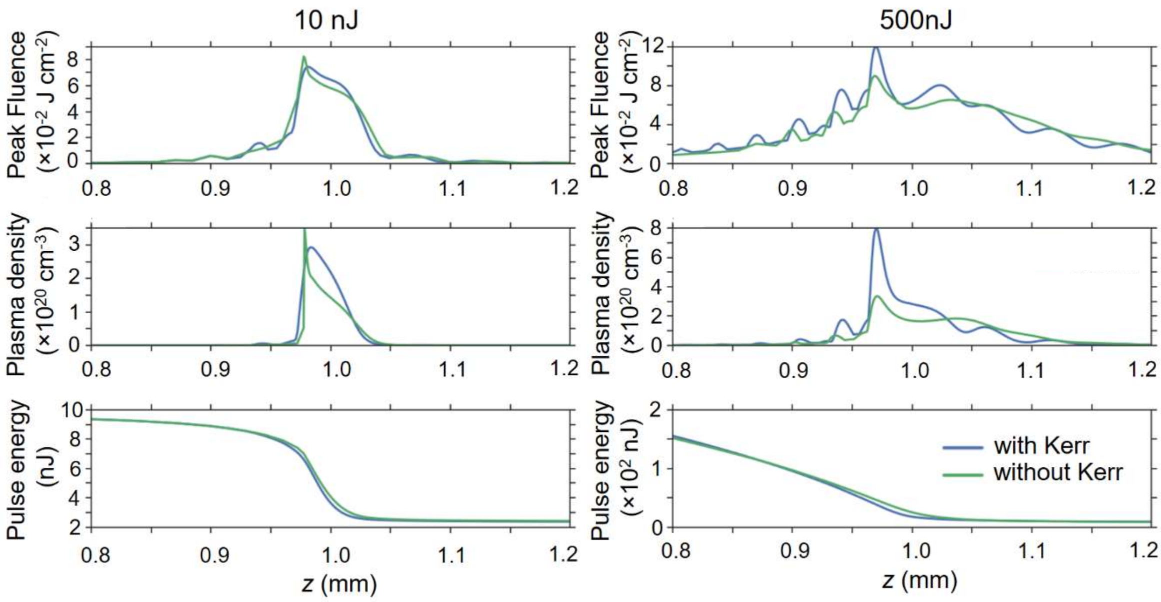

Supplementary Figure 8: Simulation results with and without the term accounting for Kerr nonlinearity. The simulations are repeated for pulses of 60-fs duration focused with a numerical aperture of 0.45 with two different energies: 10 nJ and 500 nJ. For each energy, the peak fluence, the plasma density and the pulse energy of the pulse along the optical axis are compared for simulations with (blue) and without (green) the Kerr term. The geometrical focus is positioned at z=1mm.

Two-photon absorption

An important limitation to the fluence that can be delivered in the bulk of Si is the strong depletion of the pulse energy by the highly efficient 2-photon absorption (2PA), far before reaching the focus inside Si. This is directly evidenced by plotting the pulse energy as a function of distance on the optical axis as shown in Supplementary Figure 8 (bottom). For an incoming pulse energy of 500 nJ, we note a progressive depletion of the pulse energy with propagation in Si leading to less than 20 nJ (< 4%) reaching the geometrical focus. Because the intensity gradually decreases due to divergence after the focus there is no additional absorption while propagating after the depth of 1 mm.

Plasma defocusing and absorption

Other important contributions to the observed clamping come from plasma effects. These are revealed in Supplementary Figure 9 where we compare the delivered fluence along the optical axis for simulations in which plasma effects have been switched off. In the model, plasma effects are included through the free-current term that is complex (Eq. 8). Then, we can arbitrarily set to zero its real part corresponding to plasma defocusing and/or its imaginary part, which describes losses by inverse Bremsstrahlung absorption. Compared to the original problem (all effects), we see in Supplementary Figure 9 that plasma defocusing plays an essential role near the focal region (about 50 μm before the focus) as also suggested by a recent theoretical study by Zavedeev *et al.*[17]. However, the absence of inverse Bremsstrahlung absorption is also a major screening contribution as the simulations without plasma effects show that the thresholds for modifications would be largely exceeded at 500 nJ (fluence threshold measured in section 1 and the critical plasma density (6.6×10^{20} cm^{-3}) usually taken as criterion for optical breakdown).

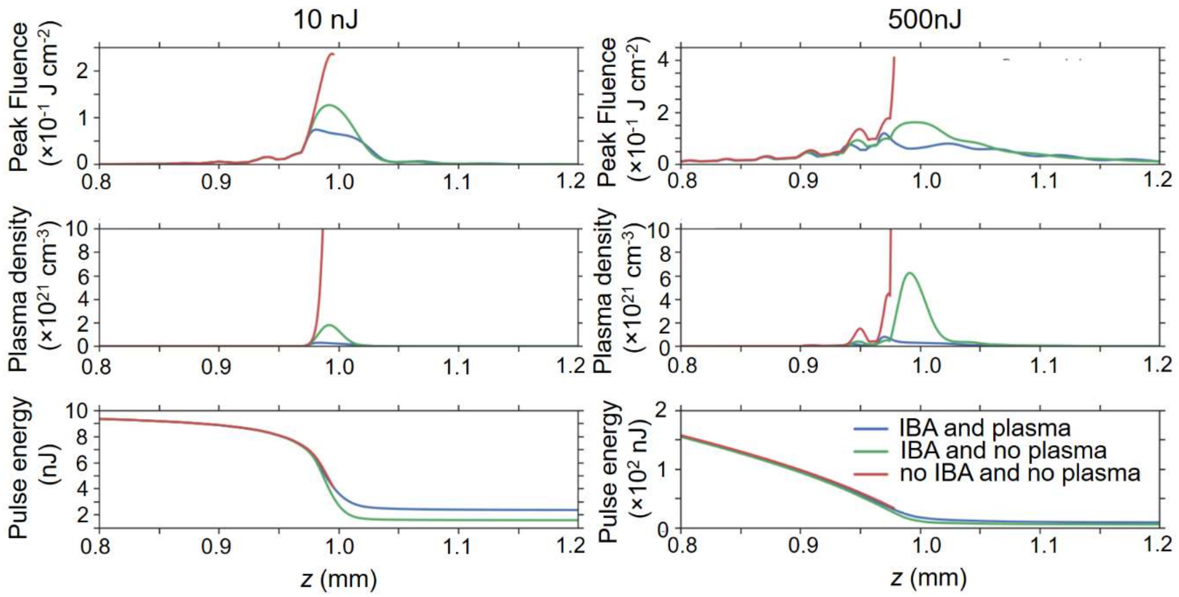

Supplementary Figure 9 | Simulation results with and without plasma effects. The simulations are repeated for pulses of 60-fs duration focused with a numerical aperture of 0.45 with two different energies: 10 nJ and 500 nJ. For each energy, the peak fluence, the plasma density and the pulse energy of the pulse along the optical axis are compared for simulations with all plasma effects (blue), with inverse Bremsstrahlung absorption but no index variation induced by the plasma (green) and without any plasma effects (red). The geometrical focus is positioned at z=1mm.

High NA cases

To overcome both limitations (2PA and plasma), a natural option is to rely on increased NA so that higher angular components contribute to the focus limiting the intensity and the created plasma density in the prefocal region. In Supplementary Figure 10, we compare simulated fluence distributions around the focus for NA=0.65 and NA=1.5. Interestingly, we see in the case of NA=1.5 that the peak fluence is twice higher and we obtain about one order of magnitude increase of the plasma density compared to NA=0.65 (not shown here). This confirms the benefit of increasing the NA but one must note that the delivered peak fluence at NA=1.5, the typical maximum NA for an experiment with oil immersion focusing, remains below the required threshold for material modification (see section 1). Also, it is usually admitted that experiments with strong focusing limit the importance of self-focusing. In our simulations we confirmed that the impact of self-focusing in the cases of 10 and 500 nJ becomes almost negligible at NA=1.5.

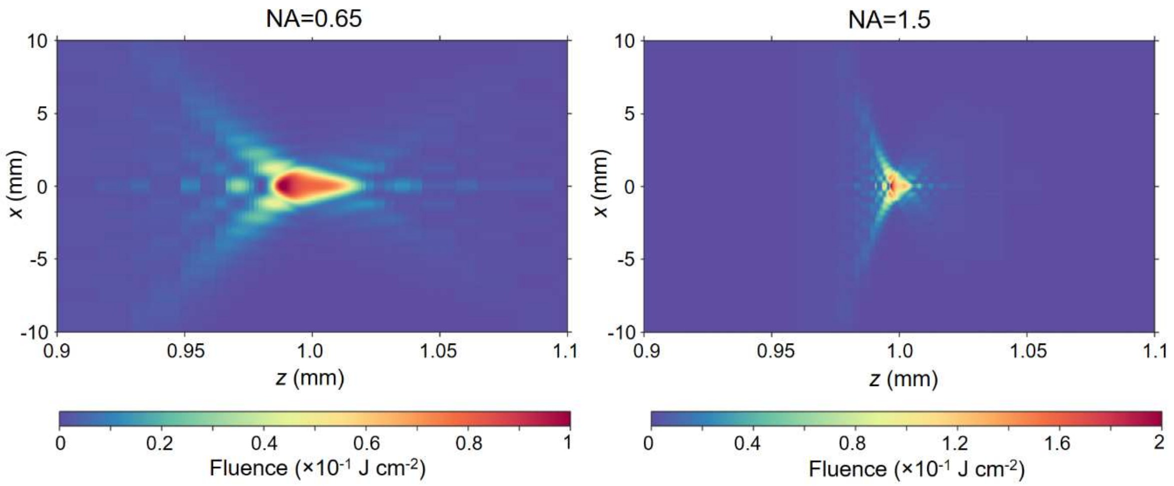

Supplementary Figure 10 | Simulated fluence distributions for High NA values. The two images show the fluence distribution delivered near the focus (cross-sections) for a low energy pulse (10 nJ) focused with NA=0.65 and NA=1.5 inside Si. The laser pulse arrives from left.

Supplementary references

1. Leyder, S. Ionisation nonlinéaire dans les matériaux diélectriques et semiconducteurs par laser femtoseconde accordable dans le proche infrarouge. *PhD Dissertation, Aix-Marseille Univ.* (2013).
2. Austin, D., Kafka, K., Blaga, C. I., Dimauro, L. F. & Chowdhury, E. Measurement of femtosecond laser damage thresholds at mid IR wavelengths. in *Proc. SPIE* **9237,** 92370V (2014).
3. von der Linde, D. & Schüler, H. Breakdown threshold and plasma formation in femtosecond laser-solid interaction. *J. Opt. Soc. Am. B* **13,** 216–222 (1996).
4. J.-C. Diels, W. Rudolph, Ultrashort laser pulse phenomena: Fundamentals, Techniques and Applications on a femtosecond time scale, (Academic Press, 1996).
5. Mero, M., Liu, J., Rudolph, W., Ristau, D. & Starke, K. Scaling laws of femtosecond laser pulse induced breakdown in oxide films. *Phys. Rev. B* **71,** 115109 (2005).
6. Kim, M.-S., Scharf, T., Etrich, C., Rockstuhl, C. & Peter, H. H. Longitudinal-differential interferometry: direct imaging of axial superluminal phase propagation. *Opt. Lett.* **37,** 305–307 (2012).
7. Mouskeftaras, A. *et al.* Direct measurement of ambipolar diffusion in bulk silicon by ultrafast infrared imaging of laser-induced microplasmas. *Appl. Phys. Lett.* **108,** 41107 (2016).
8. Safrani, A. & Abdulhalim, I. Real-time phase shift interference microscopy. *Opt. Lett.* **39,** 5220–5223 (2014).
9. Bruning, J. H. *et al.* Digital Wavefront Measuring Interferometer for Testing Optical Surfaces and Lenses. *Appl. Opt.* **13,** 2693–2703 (1974).
10. Kolesik, M. & Moloney, J. V. Nonlinear optical pulse propagation simulation: From Maxwell's to unidirectional equations. *Phys. Rev. E* **70,** 036604 (2004).
11. Fedorov, V. Y., Chanal, M., Grojo, D. & Tzortzakis, S. Accessing extreme spatio-temporal localization of high power laser radiation through transformation optics and scalar wave equations. *Phys. Rev. Lett.* **117,** 043902 (2016).
12. Li, H. H. Refractive index of Silicon and Germanium and its Wavelentght and Temperature Derivatives. *J. Phys. Chem. Ref. Data* **9,** 561–658 (1980).
13. Lin, Q. *et al.* Anisotropic nonlinear response of silicon in the near-infrared region. *Appl. Phys. Lett.* **91,** 071113 (2007).
14. Mouskeftaras, A. *et al.* Self-limited underdense microplasmas in bulk silicon induced by ultrashort laser pulses. *Appl. Phys. Lett.* **105,** 191103 (2014).
15. Pearl, S., Rotenberg, N. & Van Driel, H. M. Three photon absorption in silicon for 2300-3300 nm. *Appl. Phys. Lett.* **93,** 131102 (2008).
16. Bor, Z. Distortion of femtosecond laser pulses in lenses. *Opt. Lett.* **14,** 119–121 (1989).
17. Zavedeev, E. V., Kononenko, V. V. & Konov, V. I. Delocalization of femtosecond laser radiation in crystalline Si in the mid-IR range. *Laser Phys.* **26,** 16101 (2016).
